# Acupuncture in subjects with cold hands sensation: study protocol for a randomized controlled trial

**DOI:** 10.1186/1745-6215-15-348

**Published:** 2014-09-04

**Authors:** Jung-Chul Seo, Hyun-jong Lee, Min-Ah Kwak, Sung-Hoon Park, ImHee Shin, Woo-Sung Yun, Kihyuk Park

**Affiliations:** Comprehensive and Integrative Medicine Institute, 3056-6 Daemyeong 4-dong, Nam-gu, Daegu, 705-718 Republic of Korea; Department of Acupuncture & Moxibustion, College of Oriental Medicine, Daegu Haany University, 165 Sang-dong, Suseong-gu, Daegu, 706-060 Republic of Korea; Department of Internal Medicine, College of Oriental Medicine, Daegu Haany University, 165 Sang-dong, Suseong-gu, Daegu, 706-060 Republic of Korea; Department of Medical Statistics, School of Medicine, Catholic University of Daegu, 3056-6 Daemyeong 4-dong, Nam-gu, Daegu, 705-718 Republic of Korea; Division of Vascular Surgery, Department of sugery, School of Medicine, Catholic University of Daegu, 3056-6 Daemyeong 4-dong, Nam-gu, Daegu, 705-718 Republic of Korea

**Keywords:** Acupuncture, Cold hands sensation, Laser Doppler perfusion image

## Abstract

**Background:**

Cold hands sensation is a common disorder within the Korean population. Many Korean family physicians believe that it is a mild early manifestation of Raynaud’s phenomenon (RP), or may be related to RP. RP is characterized by reversible digital vasospasm provoked by cold temperatures and/or emotional stress, and doctors often prescribe medications that are used in treatment of RP for subjects with cold hands. However, this has not shown a clear benefit, and these medications can cause unwanted side effects. It is also reported that traditional Korean medicine, including acupuncture, is widely used to treat cold hands, although the current level of evidence for this approach is also poor and to date, there have been no published randomized controlled clinical trials (RCTs) evaluating the efficacy and safety of acupuncture for cold hands. We have therefore designed a pilot RCT to obtain information for the design of a further full-scale trial.

**Methods/Design:**

The proposed study is a five-week pilot RCT. A total of 14 subjects will be recruited and randomly allocated to two groups: an acupuncture plus medication group (experimental group) and a medication-only group (control group). All subjects will take nifedipine (5 mg once daily) and beraprost (20 mg three times daily) for three weeks. The experimental group will receive additional treatment with three acupuncture sessions per week for three weeks (nine sessions total). The primary outcome will be measured using a visual analogue scale. Secondary outcomes will be measured by blood perfusion in laser Doppler perfusion imaging of the hands, frequency and duration of episodes of cold hands, and heart rate variability. Assessments will be made at baseline and at one, three, and five weeks thereafter.

**Discussion:**

This study will provide an indication of the feasibility and a clinical foundation for a future large-scale trial.

**Trial registration:**

This study was registered at Korean Clinical Research Information Service (CRIS) registry on 5 August 2013 with the registration number #KCT0000817.

## Background

One of the normal physiological responses to cold temperatures or emotional stress is a reduction of the skin temperature to preserve body heat and maintain a normal body core temperature. Reactions characterized by excessive peripheral vasospasm may evoke clinical symptoms, and complaints of cold hands sensation are very common among the general population [1]. Cold hands sensation is defined as hands that become intolerably cold when exposed to normal temperature, in which most individuals feel no cold. There can be a confusion between cold hands sensation and Raynaud’s phenomenon (RP), as both have cold hands as a symptom. The signs of RP include cold and numb hands when exposed to cold temperatures, with subsequent pain and skin becoming blue in colour [2]. In a community-based survey of approximately 7,000 people, almost 12% responded that they had experienced unusual sensitivity to cold temperatures in their fingertips or toes [3, 4]. This may be due to a mild, early manifestation of Raynaud’s phenomenon (RP) or may be related to RP [1, 5], which is characterized by a reversible digital vasospasm provoked by cold temperatures and/or emotional stress [6]. A study by Choi *et al*. [7] found that in South Korea, 43% of patients with an abnormal cold sensation in the hands or feet had RP, and that primary RP represented 73% of cases of RP, and many family physicians prescribe medications for cold hands sensation based upon treatment recommendations for RP [8].

The exact prevalence of primary RP is unknown. It is a common disorder, especially in the general practice setting [9], and its prevalence is thought to be high compared with the low number of patients who seek treatment for it [10]. The treatment is mostly dependent on the etiology of the disorder and on the presence and severity of the individual symptoms. Lifestyle modifications including avoidance of cold exposure and caffeine, discontinuation of smoking, and sufficient body insulation, are the first line of treatment for the prevention of vasospasm [11, 12]. If these modifications are insufficient, calcium channel blockers are the most widely used pharmacological agents for treatment [4, 13, 14], and in severe forms of RP, prostaglandins, endothelin-1 receptor antagonists, and specific inhibitors of phosphodiesterase-5 are the treatments of choice [6]. Although a wide variety of medications are used for treatment of RP, none have been shown to have a clear benefit, and all may be associated with unwanted side effects including headache, flushing, and dizziness. There is currently no gold standard or universal guideline for the treatment of RP [6, 15].

In Korea, traditional treatments including acupuncture and herbal medications have been widely used to treat cold hands for many years, and there are a number of published reports regarding these treatments for cold hands [16–18]. Nonetheless, the present level of evidence is poor because of small sample sizes or short durations of these published studies. In addition, even though there are a few published randomized controlled trials (RCT) regarding acupuncture and RP [19, 20], there is no published RCT evaluating the efficacy and safety of acupuncture in subjects with cold hands sensation. The need remains for more rigorous studies that will elucidate the efficacy and safety of acupuncture for subjects with cold hands sensation. The aim of the present study was to collect preliminary data on acupuncture compared with conventional pharmaceutical agents alone for the treatment of cold hands sensation. The results of this study will provide evidence for the feasibility of this trial design as well as basic data for a large-scale RCT of acupuncture for subjects with cold hands sensation.

### Hypotheses

In this randomized controlled two-arm clinical trial, we will evaluate the acupuncture treatment as an adjunct therapy to cold hands sensation. The hypothesis is that the addition of acupuncture treatment to medication reduces the severity of cold hands sensation (measured using a visual analogue scale (VAS)) significantly more than medications alone.

## Methods/Design

### Design

The study is a randomized controlled pilot clinical trial. It is designed to obtain basic information of a further full-scale trial about acupuncture treatment in subjects with cold hands sensation. The protocols to be used adhere to the principles of the Declaration of Helsinki and have been approved by the Institutional Review Board of Daegu Catholic University Hospital (approval number IORG0004453), where the study will take place. The trial is registered with the Korean Clinical Research Information Service (CRIS) registry. Written informed consent will be obtained from each participant before any treatment is given.

**Figure 1:**
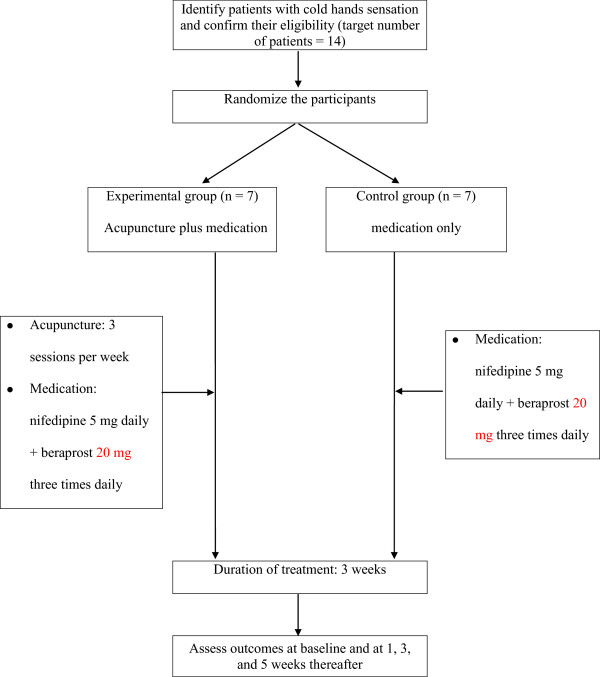
**Flow chart of the pilot randomized controlled trial.**

The outcome assessments and statistical analyses will be performed by professionals blinded to the assignment of subjects. The trial process is presented in Figure 1. The trial will run for five weeks. Subjects will be randomly allocated to one of two groups, a control group, in which subjects will receive only medication for three weeks, and an experimental group, in which subjects will receive the same medication as well as nine acupuncture sessions (three sessions per week for three weeks). Assessments will be made at baseline and again at one, three, and five weeks thereafter. The five-week assessment will be performed two weeks after treatment cessation. This study will be conducted between late November 2013 to February 2014 which is winter season in South Korea. We will record room temperature to control for temperature bias in this clinical trial.

### Recruitment

Participants will be recruited through advertisements on hospital websites and on bulletin boards. If subjects are interested in participating, they will be invited to the hospital for a screening meeting. Eligibility will be determined by a vascular surgeon based on the results of physical and photoplethysmography examinations. If eligible, subjects will be guided through the informed consent process. After written consent is obtained, a study researcher will randomly allocate each participant to one of the two treatment groups.

### Participants

A target sample size of 14 subjects with cold hands sensation has been set. One of the main objectives of this study is to provide an estimate of the sample size required for the full-scale randomized controlled clinical trial.

### Inclusion criteria

Participants must meet the following criteria for inclusion in the study: cold hands sensation or RP, photoplethysmography showing decreased arterial pulse amplitude or abnormal ischemic pulse waveform such as biphasic or monophasic wave, aged between 20 and 75 years, VAS score of >3 for cold hands sensation, symptom duration of least one month, available for follow-up during the entire trial period, and provide written informed consent.

### Exclusion criteria

Participants will be excluded from the study if they meet any of the following criteria: one or more ulcerated fingers, acute ischemic disease requiring reperfusion surgery, high bleeding tendency due to anticoagulant medications, significant renal or hepatic disease, severe psychiatric or psychological disorders, known hypersensitivity reaction to acupuncture treatment, alcohol and/or drug abuse, pregnant, lactating, or planning pregnancy, any individual deemed ineligible by a physician, refusal to participate in the trial or to provide informed consent, or an inability to comprehend or express oneself in the Korean language.

### Randomization

Subjects will be randomized using a computerized random number generator by an independent statistician who is blinded to subject assignment. The method which we use for randomization concealment is sequentially numbered, opaque sealed envelopes. Block randomization will be performed once a participant’s eligibility is confirmed and written informed consent has been obtained. Treatments will be scheduled after randomization.

### Interventions

Subjects will be randomly divided into two treatment groups: an experimental group (acupuncture plus medication group) and a control group (medication only group). The medication will be administered every day for three weeks and the acupuncture sessions performed three times per a week for three weeks (nine times in total). There are no other interventions such as moxibustion, exercises, or lifestyle advice in this study.

#### Medications

Oral nifedipine (calcium channel blocker, 5 mg once daily) and beraprost (prostaglandin analog, 20 mg three times daily) will be prescribed for all subjects in both groups.

#### Acupuncture treatment

The following acupoints will be used based on previously published work [21]: unilateral GV20, bilateral ST36, PC6, TE5, LI4, and EX-UE9 (extra points). In total, 17 acupoints will be used. Sterilized disposable acupuncture needles (DongBang Acupuncture Inc., Korea) 0.25 × 40 mm in size will be manually inserted into each of the acupoints. After needle insertion, the *deqi* sensation will be induced by manual stimulation. The needles will be inserted for 20 ± 5 minutes and then removed. Acupuncture will be practiced by Korea Medical Doctors (KMD) who are licensed by Ministry of Health and Welfare.

### Data collection

In this study, the primary outcome will be measured by VAS. The secondary outcomes are changes in microvascular blood perfusion, frequency and duration of cold hands sensation episodes, and heart rate variability (HRV) measurements. Both primary and secondary outcomes will be assessed at baseline and at one, three, and five weeks after initiation of treatment. The treatment and outcomes measurement schedules are presented in Table 1.

**Table 1:** **Schedule of treatments and outcome measurements throughout the five-week randomized controlled trial**

		Baseline	Treatment period									Follow-up period	
		Week 0	Week 1			Week 2			Week 3			Week 4	Week 5
Measurement	VAS	√	√						√				√
	Blood perfusion	√	√						√				√
	Frequency and duration of cold hands episodes	√	√						√				√
	HRV	√	√						√				√
Treatment	Medication		√			√			√				
	Acupuncture		√	√	√	√	√	√	√	√	√		

HRV: Heart rate variability, VAS: visual analogue scale.

#### Primary outcome measurements

##### VAS

The severity of cold hands sensation episodes ranges across a continuum of values but cannot easily be measured directly. Therefore, we will use a 10-cm VAS as the measurement instrument to determine the severity of cold hands sensation. Each subject will rate each episode of cold hands sensation on a scale of 0 to 10, where 0 indicates the absence of the cold hands sensation and 10 indicates the worst cold hands sensation imaginable. VAS measurements will be made at baseline and at one, three, and five weeks thereafter.

#### Secondary outcome measurements

##### Blood perfusion

Laser Doppler perfusion imaging (LDPI) with a Perimed AB LDPI system (Perimed AB, Jaerfaella, Sweden) will be used to measure the perfusion of microcirculation in the skin of the hands. The LDPI system has a moving laser beam that scans the tissue in steps and a photo detector that measures the backscattered light. The frequency shift of the light has a direct relation to the perfusion of the lighted area of skin, according to the Doppler principle [22].

The largest scanning image is 64 **×** 64 (4096) points and low, medium, and high resolution modes are available. The largest scanning area is about 20 **×** 20 cm. Measuring time is about 50 milliseconds per point, and 4 minutes for a 64 **×** 64 image. A normal photo is taken along with the blood perfusion scan in order to localize the area of interest. When the scan is completed, the region of interest is selected for analysis of the mean blood perfusion and its changes using the built-in LDPI windows 2.5 software.

##### Frequency and duration of cold hands sensation episodes

The subjects will record the frequency and duration of each episode of cold hands sensation. The average frequency and duration of attacks will be calculated weekly by dividing the total number of attacks and their duration by the number of a given weekly interval (observation period). The frequency and duration cold hands sensation episodes will be measured at baseline and at one, three, and five weeks thereafter. We will record the frequency and duration of cold hands sensation episodes by patient diaries.

##### Heart rate variation

The pathogenesis of cold hands sensation is still unclear. Neural and intravascular mechanisms are thought to be involved in the pathological progress. The role of the autonomic nervous system is most often discussed, with particular attention to sympathetic over-reactivity. HRV analysis is a reliable non-invasive test that is used assess autonomic nervous system function [23].

HRV will be measured using a Medicore SA-2000E analyzer (Medicore Co., Ltd., Seoul, South Korea) with four limb electrodes. To minimize the influence of confounding factors, all subjects were prohibited from using drugs, caffeine, tobacco, and alcohol for eight hours before HRV testing. The frequency domain methods of HRV analysis will be used. Low frequency (LF), high frequency (HF), LF:HF ratio, LF in normalized units, and HF in normalized units will be measured. LF reflects the sympathetic influence, whereas HF reflects the parasympathetic influence. The LF:HF ratio reflects the interaction between sympathetic and vagal activity. LF in normalized units and HF in normalized units are the respective values of LF and HF divided by the sum of LF plus HF and multiplied by 100; thus, the sum of LF in normalized units and HF in normalized units is 100 [24]. HRV measurements will be conducted at baseline and at one, three, and five weeks thereafter.

### Safety

The safety of this trial will be determined by red blood cell (RBC) count, hemoglobin level, platelet count, mean corpuscular volume (MCV), mean corpuscular hemoglobin (MCH) level, mean corpuscular hemoglobin concentration (MCHC), hematocrit (Hct), total white blood cell (WBC) count, erythrocyte sedimentation rate (ESR), aspartate aminotransferase (AST), alanine aminotransferase (ALT), blood urea nitrogen (BUN), and creatinine level, serum sodium level, serum potassium level, and serum chloride level. All subjects are evaluated twice, first at the screening visit and once more after the termination of acupuncture treatments. To monitor the safety of the acupuncture treatment, we will monitor the occurrence of edema, hemorrhage, and pain as adverse events.

Any reported adverse events will be recorded throughout the study and vital signs will be monitored at each visit. The subjects will be asked to voluntarily report information about adverse events, and the researcher will confirm the occurrence of adverse events through methods such as a medical interview. Details about adverse events, such as the date of occurrence, degree of severity, causal relationship with the treatment, other treatments or medications that are suspected to cause the adverse event, and treatment of the adverse event will be recorded in detail.

### Withdrawal and dropout

All subjects will have the right to withdraw from the study at any time. Participation will be ended at any stage if the subject refuses to continue, withdraws consent, or violates the inclusion or exclusion criteria or the trial protocol. The trial will be stopped if the principal investigator believes that there are unacceptable risks of serious adverse events.

### Statistical analysis

The statistical significance level will be set at 5%, and the data will be processed with the last observation carried forward method for the intention-to-treat analysis. Statistical analysis in this study will be performed using IBM SPSS Windows version 19.0 statistical software (IBM Co., Armonk, NY, USA) and will be based on the Korean Clinical Trial Statistics Guidelines.

The study will identify the comparative equivalence of demographic variables and clinical characteristics between the experimental and control groups by performing the two sample t-test or Mann-Whitney U test if normality test is satisfied or not for continuous data, chi-square test for categorical data.

A repeated measure two-factor analysis will be performed to identify differences in VAS, blood perfusion, frequency and duration of cold hands sensation attacks and HRV scores between the experimental and control group based on time (baseline, weeks one, three, and five). If the interaction between group and time is statistically significant, the point at which the pattern of results between the two groups changes will be checked using the contrast analysis. To compare groups and the incidence frequency of adverse events related to acupuncture and medication, the chi-square test will be used.

## Discussion

Cold hands sensation is a common disorder within the Korean population. In Korea, many physicians believe the phenomenon is related to RP, and they may prescribe pharmaceutical treatments similar to those used for patients with RP [5, 8]. However, there is no established guideline for RP therapy, and many of the agents most frequently used in the treatment of RP are used off-label [15] and consist mainly of vasodilators, in particular calcium channel antagonists such as nifedipine. While these agents have proven useful in some reports [14], in about 50% of patients, there is no beneficial effect [15]. Furthermore, their use has often been associated with an unacceptably high incidence of side effects including edema, flushing, erythema, dizziness, nausea, palpitations, and drowsiness [25, 26]. Beraprost is the first oral prostaglandin analog with vasodilatory and antiplatelet effects which has been used in the treatment of RP. To date, one double blind study has indicated that there was no difference between oral beraprost and placebo in the treatment of primary RP, and the same study found that patients in the beraprost group reported a significantly higher incidence of side effects including headache; dose increase was also limited by side effects such as headache, flushing, and diarrhea [15, 27]. The high prevalence of RP in the general population and the unfavorable side effects profiles of the present medications further demonstrate the need for alternative treatment options.

Acupuncture is one of the most important components of complementary and alternative medicine. It is a therapy that has been used for thousands of years in Korea. Recently, there has been increased interest in the therapeutic benefits of acupuncture [28]. In South Korea, many subjects with cold hands sensation already choose traditional Korean medicines such as acupuncture for treatment. However, thus far, there are no published controlled clinical trials investigating the efficacy and safety of acupuncture for treatment of cold hands sensation. We have therefore designed this pilot RCT to guide the design of a full-scale trial. Our study will be a first randomized controlled trial regarding cold hands sensation, but it has limitation of small sample size. We expect this pilot study to provide a clinical foundation for a future large-scale trial as well as information about the feasibility of such a trial.

## Trial status

This trial’s recruitment was finished.

## Abbreviations

HF: High frequency
HRV: Heart rate variability
LDPI: Laser doppler perfusion imaging
LF: Low frequency
RP: Raynaud’s phenomenon
VAS: Visual analogue scale.
